# Postpartum Migraine Headache Coding in Electronic Health Records of a Large Integrated Health Care System: Validation Study

**DOI:** 10.2196/42955

**Published:** 2022-11-17

**Authors:** Jiaxiao Shi, Michael J Fassett, Vicki Y Chiu, Chantal C Avila, Nehaa Khadka, Brittany Brown, Pooja Patel, Nana Mensah, Fagen Xie, Morgan R Peltier, Darios Getahun

**Affiliations:** 1 Department of Research and Evaluation Kaiser Permanente Southern California Pasadena, CA United States; 2 Department of Obstetrics and Gynecology Kaiser Permanente West Los Angeles Medical Center Los Angeles, CA United States; 3 Department of Clinical Sciences Kaiser Permanente Bernard J Tyson School of Medicine Pasadena, CA United States; 4 Department of Psychiatry Jersey Shore University Medical Center Neptune, NJ United States; 5 Department of Health Systems Science Kaiser Permanente Bernard J Tyson School of Medicine Pasadena, CA United States

**Keywords:** migraine headache, validation, diagnosis, pharmacy, postpartum, medical record, health plan, electronic health record, coding, pharmacy record, diagnostic code, EHR system

## Abstract

**Background:**

Migraine is a common neurological disorder characterized by repeated headaches of varying intensity. The prevalence and severity of migraine headaches disproportionally affects women, particularly during the postpartum period. Moreover, migraines during pregnancy have been associated with adverse maternal outcomes, including preeclampsia and postpartum stroke. However, due to the lack of a validated instrument for uniform case ascertainment on postpartum migraine headache, there is uncertainty in the reported prevalence in the literature.

**Objective:**

The aim of this study was to evaluate the completeness and accuracy of reporting postpartum migraine headache coding in a large integrated health care system’s electronic health records (EHRs) and to compare the coding quality before and after the implementation of the International Classification of Diseases, 10th revision, Clinical Modification (ICD-10-CM) codes and pharmacy records in EHRs.

**Methods:**

Medical records of 200 deliveries in all 15 Kaiser Permanente Southern California hospitals during 2 time periods, that is, January 1, 2012 through December 31, 2014 (International Classification of Diseases, 9th revision, Clinical Modification [ICD-9-CM] coding period) and January 1, 2017 through December 31, 2019 (ICD-10-CM coding period), were randomly selected from EHRs for chart review. Two trained research associates reviewed the EHRs for all 200 women for postpartum migraine headache cases documented within 1 year after delivery. Women were considered to have postpartum migraine headache if either a mention of migraine headache (yes for diagnosis) or a prescription for treatment of migraine headache (yes for pharmacy records) was noted in the electronic chart. Results from the chart abstraction served as the gold standard and were compared with corresponding diagnosis and pharmacy prescription utilization records for both ICD-9-CM and ICD-10-CM coding periods through comparisons of sensitivity, specificity, positive predictive value (PPV), negative predictive value (NPV), as well as the summary statistics of *F*-score and Youden *J* statistic (*J*). The kappa statistic (κ) for interrater reliability was calculated.

**Results:**

The overall agreement between the identification of migraine headache using diagnosis codes and pharmacy records compared to the medical record review was strong. Diagnosis coding (*F*-score=87.8%; *J*=82.5%) did better than pharmacy records (*F*-score=72.7%; *J*=57.5%) when identifying cases, but combining both of these sources of data produced much greater accuracy in the identification of postpartum migraine cases (*F*-score=96.9%; *J*=99.7%) with sensitivity, specificity, PPV, and NPV of 100%, 99.7%, 93.9%, and 100%, respectively. Results were similar across the ICD-9-CM (*F*-score=98.7%, *J*=99.9%) and ICD-10-CM coding periods (*F*-score=94.9%; *J*=99.6%). The interrater reliability between the 2 research associates for postpartum migraine headache was 100%.

**Conclusions:**

Neither diagnostic codes nor pharmacy records alone are sufficient for identifying postpartum migraine cases reliably, but when used together, they are quite reliable. The completeness of the data remained similar after the implementation of the ICD-10-CM coding in the EHR system.

## Introduction

Migraine is a common neurological disorder characterized by repeated headaches of varying intensity [1]. Often presenting in one side of the head, these headaches are typically accompanied by nausea, vomiting, and extreme sensitivity or intolerance to light and sound that lasts for 4 to 72 hours [1,2]. Globally, migraines were ranked as the sixth most prevalent cause of diseases [3] and the third cause of disabilities [4]. The prevalence of migraine increases initially with age, peaks at about 30-39 years, and then gradually declines over time [3]. Migraine headaches are more prevalent among women of reproductive ages (15-49 years), with 25% of women affected worldwide [3]. Women also experience more severe symptoms with a longer duration of attack, recurrent headaches, and higher migraine-related disability than men [5]. Moreover, migraines during pregnancy have been linked with preeclampsia or postpartum stroke [6,7]. Migraines during the postpartum period are particularly common, with reported prevalence rates ranging from 5% to 55% [8]. However, due to the lack of a validated instrument for uniform case ascertainment, there is uncertainty in the reported prevalence in the literature.

In 2009, the Health Information Technology for Economic and Clinical Health (HITECH) Act was enacted to promote the adoption of electronic health record (EHR) systems among health care providers [9]. Consequently, many health care institutions implemented EHR systems in their facilities [10]. The Kaiser Permanente Southern California (KPSC) integrates EHRs with comprehensive inpatient and outpatient clinical records, and prescription medication history can be used for pharmacoepidemiologic research, including examining potential risk factors and triggers for postpartum migraine and its effects on the lives of the affected individuals.

Currently, many health care systems use the official International Classification of Diseases-Clinical Modification (ICD-CM) coding systems to classify diagnoses and procedures in the EHR [11]. In KPSC settings, ICD-9-CM coding was transitioned to the ICD-10-CM coding system after October 1, 2015, which provides increased specificity and details for many health conditions [11]. However, the accuracy of postpartum migraine headache data in the EHRs, including the impact of the ICD-9-CM/ICD-10-CM transition, has not been fully examined, leaving uncertainty around the validity of case ascertainment methods. Furthermore, whether such accuracy improves when postpartum migraine headache diagnosis (Dx) codes are used in conjunction with automated pharmacy records (Rx) has not been elucidated. Therefore, we evaluated the reliability and accuracy of postpartum migraine headache Dx codes with and without the supplemental use of Rx and whether the ICD-10-CM coding system has improved or decreased the accuracy of postpartum migraine headache case ascertainment.

## Methods

### Study Setting

This study was conducted using EHR data from KPSC. The KPSC health care system comprises over 4.8 million members, 15 hospitals, and 236 medical offices throughout southern California. Prenatal and postnatal care to the member patients is provided as outpatient care at KPSC. Although most members receive their care at KPSC hospitals and <10% utilize contracting hospitals, all diagnostic, procedural, and Rx data are captured and maintained by the KPSC EHR since its full implementation in 2008. Furthermore, the characteristics of KPSC members closely reflect the Californian population [12].

### Ethics Approval

This study was approved by the KPSC institutional review board (approval 13114), and informed consent was waived, as the study was low risk and strictly involved the use of internal EHR data, that is, access permission was given to authorized personnel only when needed.

### Cohort and Sample Selection

Data were obtained retrospectively from women who delivered live infants at the KPSC health system during 2 distinct time periods: (1) January 1, 2012 to December 31, 2014 (ICD-9-CM period) and (2) January 1, 2017 to December 31, 2019 (ICD-10-CM period). We carefully selected the 2 time periods to investigate the medical coding accuracy before and after the implementation of ICD-10-CM in the KPSC system. For each time period, we randomly sampled 25 cases in each of the following 4 strata based on EHRs: (1) those without any Dx codes or Rx for migraine headaches (neither Dx nor Rx), (2) those with only Dx codes for migraine headaches (Dx only), (3) those with only Rx selected a priori as a treatment for migraine headaches (Rx only), and (4) those with both Dx and Rx (Dx+Rx). Thus, a total of 200 individual deliveries were selected for this validation study via the stratified sampling scheme mentioned above. The accuracy for each of the 4 strata, either case ascertainment (Dx only, Rx only, or Dx+Rx) or noncase ascertainment (neither Dx nor Rx), was expected to be around 85%. A sample size of 25 would provide less than 15% one-sided margin for a 90% CI of the accuracy for each stratum.

### Defining Postpartum Migraine Headache

In this study, postpartum headache was defined based on documented clinical records within the first 12 months after delivery. In particular, primary clinical Dx codes, coded by medical coders from the clinical data management team (See Multimedia Appendix 1 for ICD-9-CM/ICD-10-CM codes) and pharmacy dispense records from all inpatient and outpatient services during the postpartum periods of the index pregnancy were used to ascertain its diagnosis (see Multimedia Appendix 1 for the medication list).

### Chart Abstraction Process

Two trained research associates reviewed EHRs for documentation of a diagnosis or medication for migraine headache during the postpartum period. Women were considered to have postpartum migraine headache if either a mention of migraine headache or a prescription for the treatment of migraine headache was noted in the chart during the first year of the postpartum period. Research associates confirmed any medical diagnosis (yes/no) and prescription for treatment (yes/no) of migraine headaches for all 200 women. To ensure data quality and consistency of chart reviews between the 2 abstractors, interrater reliability assessment was performed on randomly selected charts stratified by the 4 strata (DX only, Rx only, Dx+Rx, and neither Dx nor Rx). Discrepant cases in clinical utilizations were adjudicated by the study investigators with clinical expertise (MJF and DG). The postpartum migraine headache cases abstracted through this process served as the gold standard.

### Maternal Characteristics

Maternal characteristics for KPSC births included maternal age (<20, 20-29, 30-34, and ≥35 years), race/ethnicity (categorized as non-Hispanic White [hereafter referred to as White], non-Hispanic Black [hereafter referred to as Black], Hispanic, Asian/Pacific Islander, and others/unknown), educational attainment (less than high school, high school graduate, some college, bachelor’s/associate degree, and master’s degree or higher), household income in USD (<$30,000, $30,000-$49,999, $50,000-$69,999, $70,000-$89,999, and ≥$90,000), timing of prenatal care (early or first trimester and none or late initiation), and self-reported smoking (yes/no). Gestational age at birth, reported in completed weeks, was derived from clinical estimates.

We obtained the characteristics of all births of State of California residents during the same time periods by using publicly available data that has been posted on the Centers for Disease Control and Prevention Wonder website [13]. Both the KPSC EHR and the Centers for Disease Control and Prevention Wonder website provided information on maternal characteristics, including maternal age, race/ethnicity, educational attainment, timing of prenatal care, smoking during pregnancy, and gestational age at delivery (in completed weeks of gestation). Data on median household income was estimated based on census tracts for KPSC deliveries.

### Statistical Analysis

We described the characteristics of our study population, all women who delivered in KPSC hospitals, and the State of California residents with live births during 2012-2014 and 2017-2019 by using frequency distributions. We also calculated the kappa statistic (κ), which estimates agreement between the 2 abstractors. As mentioned above, abstracted chart reviews for migraine headache cases were set as the gold standard. We compared findings from the manual chart review with corresponding diagnosis and prescription medication utilization records for ICD-9-CM and ICD-10-CM coding periods through sensitivity, specificity, positive predictive value (PPV), and negative predictive value (NPV). These performance measurements were reported as weighted percentages with corresponding 95% CIs using normalized sampling weights (*W_i_*, *i*=1,2,3,4), derived by dividing the number of deliveries in each stratum by the multiplication of the total study population and the number of samples from the corresponding stratum. To evaluate the overall performance, we also reported the summary statistics of *F*-score and Youden *J* statistic (*J*), which are composite measurements of sensitivity and PPV (*F*-score) or sensitivity and specificity (*J*). All analyses were conducted using SAS statistical software version 9.4 (SAS Institute Inc).

## Results

An overview of the patient characteristics for our sample, study population, and California state birth population is shown in Table 1. Overall, 157,501 deliveries from all KPSC hospitals were obtained during the 2 periods, with 72,471 for 2012-2014 and 85,030 for 2017-2019. Compared with California State birth data, KPSC deliveries had slightly higher percentages for maternal age over 30 years (1,465,998/2,874,396, 51% vs 96,157/157,501, 61.05%, respectively), education with college and above (1,605,882/2,874,396, 55.87% vs 114,297/157,501, 72.57%, respectively), and early prenatal care initiation (first trimester; 2,386,232/2,874,396, 83.02% vs 147,017/157,501, 93.34%, respectively). There were some discrepancies for race/ethnicity, likely due to the unknown information for 30.07% (864,311/2,847,396) of the state population versus only 3.67% (5784/157,501) for the KPSC population. Despite the stratified sampling, our sample of 200 deliveries was broadly representative of the KPSC study population with respect to maternal age, early prenatal care initiation, prenatal smoking status, and gestational age. However, Hispanics and lower household income populations (<US $69,999) were slightly oversampled.

**Table 1:** Characteristics of the women who delivered in all Kaiser Permanente Southern California hospitals and in the State of California (2012-2014 and 2017-2019).

Characteristics			Charts reviewed (n=200)^a^, n (%)		Study population (N=157,501), n (%)		California state (N=2,874,396)^b^, n (%)
**Maternal age (years)**							
	<20	6 (3)		4665 (2.96)		1,44,945 (5.04)	
	20-29	76 (38)		56,679 (35.99)		1,263,453 (43.96)	
	30-34	71 (35.50)		54,810 (34.80)		843,010 (29.33)	
	≥35	47 (23.50)		41,347 (26.25)		622,988 (21.67)	
**Race/ethnicity**							
	White	39 (19.50)		39,219 (24.90)		372,037 (12.94)	
	Black	15 (7.50)		10,862 (6.90)		68,195 (2.37)	
	Hispanic	118 (59)		78,853 (50.07)		1,356,354 (47.19)	
	Asian/Pacific Islander	25 (12.50)		22,783 (14.47)		213,499 (7.43)	
	Others/unknown	3 (1.50)		5784 (3.67)		864,311 (30.07)	
**Educational attainment**							
	Less than high school	5 (2.50)		4355 (2.77)		435,360 (15.15)	
	High school graduate	49 (24.50)		35,411 (22.48)		694,118 (24.15)	
	Some college	54 (27)		32,616 (20.71)		558,288 (19.42)	
	Bachelor’s/associate degree	61 (30.50)		54,293 (34.47)		729,896 (25.39)	
	Master’s degree/above	27 (13.50)		27,388 (17.39)		317,698(11.05)	
	Missing	4 (2)		3438 (2.18)		139,036 (4.84)	
**Household income (USD)^c^**							
	<$30,000	3 (1.50)		5194 (3.30)		N/A^d^	
	$30,000-$49,999	50 (25)		39,969 (25.38)		N/A	
	$50,000-$69,999	73 (36.50)		47,864 (30.39)		N/A	
	$70,000-$89,999	45 (22.50)		32,486 (20.63)		N/A	
	≥$90,000	29 (14.50)		31,925 (20.27)		N/A	
	Missing	0 (0)		63 (0.04)		N/A	
**Timing of prenatal care**							
	First trimester	186 (93)		147,017 (93.34)		2,386,232 (83.02)	
	No or late care	14 (7)		9860 (6.26)		442,493 (15.39)	
	Missing	0 (0)		624 (0.40)		45,671 (1.59)	
	Smoking during pregnancy	8 (4)		6420 (4.08)		46,977 (1.63)	
**Gestational age (weeks)**							
	<34	10 (5)		3412 (2.17)		66,099 (2.30)	
	34-36	18 (9)		9865 (6.26)		180,352 (6.27)	
	37+	171 (85.50)		144,192 (91.55)		2,624,620 (91.31)	
	Missing	1 (0.50)		32 (0.02)		3325 (0.12)	

^a^Sample is based on data from Kaiser Permanente Southern California electronic health records of 2012-2014 and 2017-2019.

^b^Data from the natality information of Centers for Disease Control and Prevention webpage [13] (accessed on January 9, 2022).

^c^Household income is not available for the California state data.

^d^N/A: not applicable.

The overall κ between our abstractors was 100%. Table 2 gives the distribution and their sample sizes for the 4 strata (Dx+Rx, Dx only, Rx only, and neither Dx nor Rx) among our overall study population and by the 2 time periods. The majority (150,801/157,501, 95.75%) of the women did not have any record of diagnosis or pharmacy usage, while only 1131 (0.72%) women had Rx indicating obtaining prescription medication but without a diagnosis of postpartum migraine headache. Regardless of the uneven numbers among the 4 strata, we sampled 50 cases from each stratum. The corresponding chart review results and the normalized weights are provided in Table 2. For those with both Dx codes and Rx, our chart abstractors confirmed all 50 had a postpartum migraine headache, and those with neither were all confirmed to be noncases. The sample with postpartum migraine headache Dx codes only had 88% (44/50) of true positives, while having prescription Rx only resulted in 96% (48/50) of true positives.

Based on these comparisons against the chart review results, the performance measurements of our EHR are shown in Table 3. The overall weighted sensitivity, specificity, PPV, and NPV for postpartum migraine headaches were 100%, 99.7%, 93.9%, and 100%, respectively, if the postpartum migraine cases were determined by having either Dx or Rx. The corresponding *F*-score and Youden *J* statistic were 96.9% and 99.7%, respectively. Compared with using Dx codes alone (either Dx+Rx or Dx only) for postpartum migraine case identification, using Rx alone (either Dx+Rx or Rx only) had poorer performance, especially on the sensitivity (82.7% for Dx codes alone vs 57.5% for Rx alone). Results were similar across the ICD-9-CM and ICD-10-CM coding time periods.

**Table 2:** Frequencies of the study population and chart review results by diagnosis codes and pharmacy records.

Postpartum migraine headache case ascertainment method	Study population				Sample		
	Overall (N=157,501), n (%)	2012-2014 (n=72,471), n (%)	2017-2019 (n=85,030), n (%)	Charts (n=200)		True cases by chart review (n=142), n (%)	Normalized sampling weight
Diagnosis codes + pharmacy records	2531 (1.61)	1261 (1.74)	1270 (1.49)	50		50 (100)	0.000321
Diagnosis codes only	3038 (1.93)	1488 (2.05)	1550 (1.82)	50		44 (88)	0.000386
Pharmacy records only	1131 (0.72)	393 (0.54)	738 (0.87)	50		48 (96)	0.000144
Neither diagnosis codes nor pharmacy records	150,801 (95.75)	69,329 (95.66)	81,472 (95.82)	50		0 (0)	0.019149

**Table 3:** Weighted performance measurements for postpartum migraine headache based on data sources before and after implementation of the International Classification of Diseases, 10th revision, Clinical Modification codes in the Kaiser Permanente Southern California system in 2015 (n=200).

Weighted performance measurements (%)	Overall				2012-2014				2017-2019		
	ICD-CM-9/10^a^ Dx^b^	Rx^c^	ICD-CM-9/10 Dx or Rx	ICD-CM-9/10 Dx		Rx	ICD-CM-9/10 Dx or Rx	ICD-CM-9/10 Dx		Rx	ICD-CM-9/10 Dx or Rx
Sensitivity (95% CI)	82.7 (77.7-87.8)	57.5 (48.5-66.5)	100 (100-100)	83.4 (76.5-90.3)		55.4 (42.7-68)	100 (100-100)	82 (74.6-89.5)		59.8 (46.7-72.9)	100 (100-100)
Specificity (95% CI)	99.8 (99.6-100)	100 (99.9-100)	99.7 (99.5-99.9)	99.9 (99.8-100)		100 (99.9-100)	99.9 (99.7-100)	99.6 (99.2-100)		100 (99.9-100)	99.6 (99.2-100)
PPV^d^ (95% CI)	93.5 (88.3-98.6)	98.8 (97-100)	93.9 (89.5-98.2)	97.8 (93.5-100)		98.8 (96.3-100)	97.5 (93.7-100)	89.1 (79.8-98.3)		98.8 (96.3-100)	90.3 (82.5-98.1)
NPV^e^ (95% CI)	99.3 (99-99.6)	98.3 (97.6,99)	100 (100-100)	99.3 (98.9-99.7)		98.1 (97-99.2)	100 (100-100)	99.3 (98.9-99.7)		98.4 (97.5-99.4)	100 (100-100)
*F*-score^f^	87.8	72.7	96.9	90		71	98.7	85.4		74.5	94.9
Youden *J* statistic^f^	82.5	57.5	99.7	83.3		55.4	99.9	81.6		59.8	99.6

^a^ICD-CM-9/10: International Classification of Diseases, Clinical Modification, 9th/10th revision.

^b^Dx: diagnosis codes.

^c^Rx: pharmacy records.

^d^PPV: positive predictive value.

^e^NPV: negative predictive value.

^f^*F*-score and Youden *J* statistic are statistics that capture the overall performance of a dichotomous diagnostic test.

## Discussion

This validation study was performed to determine the accuracy of identifying postpartum migraine headache cases by using data abstracted from the EHR of a large health care system with a sociodemographically diverse patient population. To our knowledge, the accuracy of the data on postpartum migraine headache using both Dx and Rx has not been validated in EHR data or the extent that the transition of ICD-9-CM to the ICD-10-CM coding system has impacted postpartum migraine headache case ascertainment. Our study showed that having either Dx or Rx had high sensitivity (100%) and specificity (99.7%) for case ascertainment. The overall performance measured by the *F*-score and Youden *J* statistic were 96.9% and 99.7%, respectively. Such findings were similar across the ICD-9-CM and ICD-10-CM coding time periods.

Over the past few decades, EHRs have become important data sources for pharmacoepidemiologic studies and have become standard among health care providers as part of the American Recovery and Reinvestment Act of 2009 (specifically, the HITECH Act) [9]. Although the EHR is a highly sophisticated information management and care delivery system that ensures quality care by providing access to comprehensive patient information and the latest best practice research, its completeness and reliability for pharmacoepidemiologic studies have been questioned due to various reasons, including clinical knowledge, attention to details, communication between providers and coders, coding procedures, and others [14-16]. At KPSC, the process of coding and coding rules of the medical diagnosis recorded in patients’ EHRs is carried out by highly skilled medical coders from KPSC’s clinical data management team. Furthermore, the individual medical coder’s accuracy has been evaluated critically for consistency. Nevertheless, we may still need to develop reliable disease-specific algorithms by using diagnosis coding in combination with other EHRs, including but not limited to pharmacy and utilization records [17]. Therefore, we performed this validation study to evaluate (1) the accuracy of postpartum migraine headache case identification in EHRs and (2) the impact of implementing the ICD-10-CM coding system in extracting data on patients with postpartum migraine headache in the health system. The findings of this study suggested that the validity of EHR data for the identification of postpartum migraine headache cases differed depending on the EHR data sources (Dx and Rx). The accuracy of case ascertainment based on Dx was higher than that of case ascertainment based on Rx in this study. We speculate that the low performance of pharmacy data on case ascertainment may be driven by lactating patients declining migraine medication to avoid infant exposure through breastmilk. Others may have opted to use over-the-counter pain medications rather than prescription medications. It is also possible that a given prescription may be an appropriate drug for conditions other than migraine. However, postpartum migraine headache cases can be identified with high accuracy if Dx codes and prescription medications for the conditions are used together. Furthermore, the transition from the ICD-9-CM coding system to the ICD-10-CM coding system had minimal impact on the overall accuracy of ascertaining postpartum migraine headache cases.

The main strength of this study is the development of a valid and reliable method of postpartum migraine headache case ascertainment by using EHR data extracted from a large insured and socioeconomically diverse southern California population, which is likely generalizable to other health care settings with similar EHR database systems. In addition, in this study, the review process includes the entire medical health record, not limited to clinical Dx codes and prescription Rx, for determining the accuracy of postpartum migraine headache. A potential limitation of this study was the use of medical record abstractors who were not blind to the source of the data. Although it is possible that this could have biased the study in unforeseen ways, a previous study that evaluated the agreement between masked and unmasked medical records abstractors reported no impact of this [18].

In conclusion, our findings suggest that postpartum migraine headache is not reliably coded in the EHRs. The use of Rx along with clinical Dx codes improves the identification of true postpartum migraine cases more than the use of clinical Dx codes alone, and the transition to the ICD-10-CM diagnosis coding system had no impact.

## Appendix

Multimedia Appendix 1 Diagnosis codes and medication list for identifying postpartum migraine headache.

## Abbreviations

Dx: diagnosis
EHR: electronic health record
HITECH: Health Information Technology for Economic and Clinical Health
ICD-9-CM: International Classification of Diseases, 9th revision, Clinical Modification
ICD-10-CM: International Classification of Diseases, 10th revision, Clinical Modification
KPSC: Kaiser Permanente Southern California
NPV: negative predictive value
PPV: positive predictive value
Rx: pharmacy records
