# Supplementary material for: Postpartum Migraine Headache Coding in Electronic Health Records of a Large Integrated Health Care System: Validation Study
Source: JMIR Form Res. 2022 Nov 17;6(11):e42955. doi: 10.2196/42955 (PMC9716418; doi:10.2196/42955)
Supplement: Multimedia Appendix 1 [file formative_v6i11e42955_app1.docx]

**Supplemental Table 1**. Diagnosis Codes and Medication List for Identifying Postpartum Migraine Headache.

| **Postpartum Migraine Headache Diagnosis Code** | **ICD-9** | 346.00, 346.01, 346.02, 346.10, 346.11, 346.12, 346.20, 346.22, 346.23, 346.30, 346.31, 346.40, 346.42, 346.50, 346.63, 346.70, 346.71, 346.72, 346.80, 346.81, 346.82, 346.83, 346.90, 346.91, 346.92, 346.93 |
| --- | --- | --- |
|  | **ICD-10** | G43.001, G43.009, G43.019, G43.101, G43.109, G43.111, G43.119, G43.409, G43.419, G43.509, G43.519, G43.701, G43.709, G43.719, G43.801, G43.809, G43.819, G43.829, G43.839, G43.901, G43.909, G43.911, G43.919, G43.B0, G43.C0, G43.D0 |
| **Postpartum Migraine Headache Medication** | | Sumatriptan, Zolmitriptan, Almotriptan Malate, Dihydroergotamine, Eletriptan, Erenumab-aooe, Ergotamine, Ergotamine Tartrate, Fremanezumab-vfrm, Frovatriptan, Galcanezumab-gnlm, Naratriptan, Rimegepant. |
